# Establishment and Reliability of an Automatic Measurement Method of Pectus Excavatum Indices Using a Deep Learning Model

**DOI:** 10.7759/cureus.84976

**Published:** 2025-05-28

**Authors:** Xicheng Deng, Siping He, Jiayi Lin, Chenhan Wang, Songxian Xie, Shanshan Hu, Kefeng Ling, Frank-Martin Haecker, Shuangquan Qu

**Affiliations:** 1 Department of Cardiothoracic Surgery, The Affiliated Children's Hospital of Xiangya School of Medicine, Central South University, Hunan Children's Hospital, Changsha, CHN; 2 Department of Radiology, The Affiliated Children's Hospital of Xiangya School of Medicine, Central South University, Hunan Children's Hospital, Changsha, CHN; 3 School of Engineering Science, Shandong Xiehe University, Jinan, CHN; 4 Data and Information Management Center, The Affiliated Children's Hospital of Xiangya School of Medicine, Central South University, Hunan Children's Hospital, Changsha, CHN; 5 Department of Pediatric Surgery, Children's Hospital of Eastern Switzerland, St. Gallen, CHE; 6 Department of Anesthesiology, The Affiliated Children's Hospital of Xiangya School of Medicine, Central South University, Hunan Children's Hospital, Changsha, CHN

**Keywords:** asymmetry index, correction index, deep learning, haller index, pectus excavatum, u-net

## Abstract

Objective: This study aimed to evaluate the consistency and accuracy of pectus excavatum (PE) indices assessment by comparing U-Net-based automated segmentation with manual measurements, aiming to reduce interobserver variability and standardize clinical workflow in PE severity evaluation.

Methods: An automatic measurement model was developed using U-Net architecture, trained on 550 chest computed tomography (CT) scans from 94 patients and validated on 164 independent scans. The model calculated three key indices (Haller, correction, and asymmetry), compared against measurements by four observers.

Results: Manual measurements showed an initial error rate of 15.9% (first three observers), reduced to 4.1% after consensus correction (p<0.01). The U-Net model exhibited stable error rates (8.7% vs 8.5% pre-/post-correction, p=0.91). Strong agreement was observed between automated and corrected manual measurements: Haller index (intra-class correlation (ICC)=0.83), correction index (ICC=0.86), asymmetry index (ICC=0.92) (all p<0.01). Bland-Altman analysis confirmed minimal bias.

Conclusion: U-Net-based automation provides reliable measurement of PE severity indices, demonstrating the potential to reduce observer-dependent variability and enhance clinical workflow efficiency. Multi-center validation is warranted to support broader radiologic applications.

## Introduction

Pectus excavatum (PE) represents the most common congenital chest wall defect and may result in impaired cardiac and pulmonary function, especially in severe cases [1]. Reliable diagnostic work-up and assessment are mandatory. The Haller index, widely used as a gold standard for the assessment of PE severity, is defined as the transverse diameter of the chest at the lowest point of the depression divided by the distance from the lowest point of the depression to the front of the vertebral body. The severity of PE is categorized as follows: <3.2 mild, 3.2-3.5 moderate, and severe >3.5 [2]. Other indices, like the correction index, are reported to evaluate more details than the Haller index [3]. Regardless of which index will be applied, the most commonly used imaging technique is a computed tomography (CT) scan to assess the PE patient. Currently, all these measurements are performed manually, suggesting inevitable interobserver variation and errors. Recently, artificial intelligence has been gradually used in clinical settings and has proven efficient and accurate. More and more artificial intelligence technologies, exemplified by deep learning, have been applied to various medical image segmentation problems such as skin lesion [4], mitotic event [5], cardiac MR image [6], and myocardium [7]. Some studies have shown the potential of deep learning or computer-aided design techniques in pectus deformity, showing the feasibility of automatic diagnosis of PE with various methods [8]. Our study aimed to use U-Net, one of the main segmentation methods [9] using a deep learning network, to conduct chest wall segmentation on CT images. With the resulting chest wall contour, a variety of indices, including Haller index, correction index, and asymmetry index, can be measured automatically. The consistency of results measured using U-Net was evaluated by comparing them with manual measurements. The current manual measurement of PE indices is time-consuming and prone to interobserver variability, particularly in selecting axial slices and landmark placement. Automating this process could not only standardize assessments across institutions but also expedite clinical workflows, enabling rapid preoperative planning and postoperative follow-up. This study aims to validate a U-Net-based method for automated PE index calculation, with direct implications for improving reproducibility in multi-center studies and routine radiological practice.

## Materials and methods

Patients and CT images

After approval of the institutional review board at our hospital, a retrospective study was conducted. The data set included continuous chest CT images and thoracic spine CT images (n=9400). Records were randomly selected from patients with pectus excavatum admitted from January 2015 to December 2019, who had undergone at least one chest CT before pectus repair and were reviewed. The sagittal and coronal CT images (n=5640) were excluded. Those with blurred sternum contour (n=3210) were also excluded, which may affect segmentation and index calculation. The final chest CT images (n=550) were obtained from 94 pectus excavatum patients (female 16/94, median age 6.6 (interim 4.4-9.6) years old), including mild pectus excavatum (n=117), moderate pectus excavatum (n=86), and severe pectus excavatum (n=347). All chest CT images were extracted from the picture archiving and communication system (PACS) for deep learning U-Net model training. For model test and performance comparison, chest CT images of another 164 patients (female 30/164, median age 7.5 (interquartile 5.9-10.4) years old) were extracted and measured both manually and using the U-Net model.

Computed tomography examination

All computed tomography examinations were performed under the following standard conditions: Scanning technology: Philips CT Aura spiral CT scanning (Philips, Hamburg, Germany) and computer image post-processing workstation. The scanning technical parameters: voltage 110-120 kV, current 150 mA, layer thickness and interval 5-7 mm. Subjects were in the supine position, awake, or sedated with oral administration of 10% chloral hydrate, 0.2-0.4 ml/kg as appropriate.

Data pre-processing

With data enhancement, the variability of the training dataset can be increased, and the overfitting degree in the training process can be alleviated. The steps include: (1) image rotation, rotation angle is from -25 to 25, interval 5; (2) image scale transformation, scaling ratio is (0.85, 1.15), interval is 0.05; (3) flip, flip the image horizontally and vertically. When expanding the CT image, the corresponding annotation also needs the same transformation. After data enhancement, the amount of training data is expanded from 550 to 11000, which is 20 times the original. All images were resampled to a resolution of 256 × 256 pixels before deep learning.

U-Net introduction

**Figure 1:**
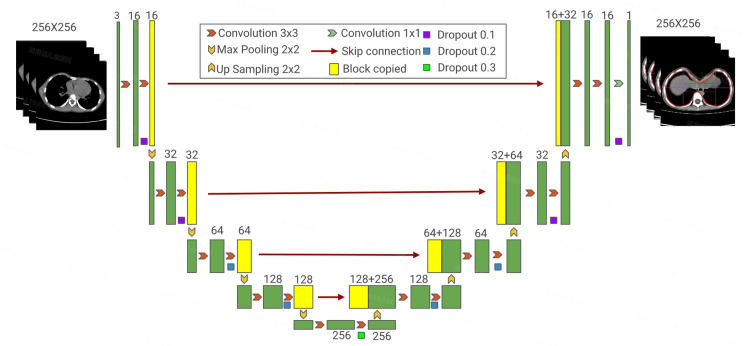
U-Net architecture for automated segmentation in pectus excavatum measurement.

In this paper, the U-Net network, one of the most popular deep learning models, was chosen to assist in the measurement of pectus excavatum indices. U-Net is an end-to-end encoder-decoder neural network trained to segment images automatically. The network consists of a contracting path (in the encoder part) and an expansive path (in the decoder part), which gives it the U-shaped architecture. The encoder comprises a series of convolutional layers, each layer followed by a nonlinear activation function and a max pooling operation. Correspondingly, the decoder part consists of a sequence of up-convolutions. It combines the feature and spatial information extracted by the encoder and concatenations of high-resolution features from the contracting path with skip connections. Each layer of the contracting path consists of two 3×3 convolution layers, one rectified linear unit (ReLU), and one max pooling layer. In the expansive path, every step includes one 2×2 up-convolution layer, one concatenation operation with the related feature map by skipped connections, and two 3×3 convolution layers. Overall, U-net is a deep neural network that has 23 layers (Figure 1).

U-Net loss function

During the whole training period of a convolutional neural network (CNN) model, the parameters are optimized with gradient descent approaches based on the errors calculated by a loss function, which compares the forward predictions and ground truth results. The loss function is critical for network optimization. Cross-entropy (CE) and the Dice coefficient (DC) have typically been used for image segmentation [10]. Despite the recent progress of using CNNs for medical image segmentation, the commonly used loss functions generally evaluate pixel-wise similarity. Especially, CE and DC focus on extracted features from specific regions of the whole image. While this can result in good classification and segmentation performance, local resultant loss function values may not really correspond to a meaningful segmentation. Any noisy result can cause many unnecessary contours in the background, representing incorrect segmentation, and object boundaries can be fuzzy due to the difficulty of classifying pixels near the boundary. In this paper, we adopted an active contour loss function inspired by the general idea of active contour model building in region and length terms for medical image segmentation by U-Net-like based deep learning architectures, which can lead to more precise chest wall contour segmentation because the geometrical information is taken into consideration.

Model training

We implemented our networks using Keras 1.1.0 (developed by François Chollet, maintained by the Google Brain team, Mountain View, CA) with Tensorflow_GPU 1.10 (Google Brain team, Mountain View, CA) as the backend, leveraging CUDA 10.0 and cuDNN v7.4 (NVIDIA Corporation, Santa Clara, CA) for GPU acceleration. The training was performed on a workstation equipped with an Intel Core i7-8700K CPU (six cores/12 threads) and an NVIDIA GeForce RTX 2080 Ti GPU (4352 CUDA cores, 11GB GDDR6 VRAM, 13.4 TFLOPS FP32 performance). Models were trained until convergence using the ADAM optimizer with a learning rate of 10^−4^. All axial CT image sets used for training were manually labeled as ground truth segmentation masks.

Automatic measurement of indices

**Figure 2:**
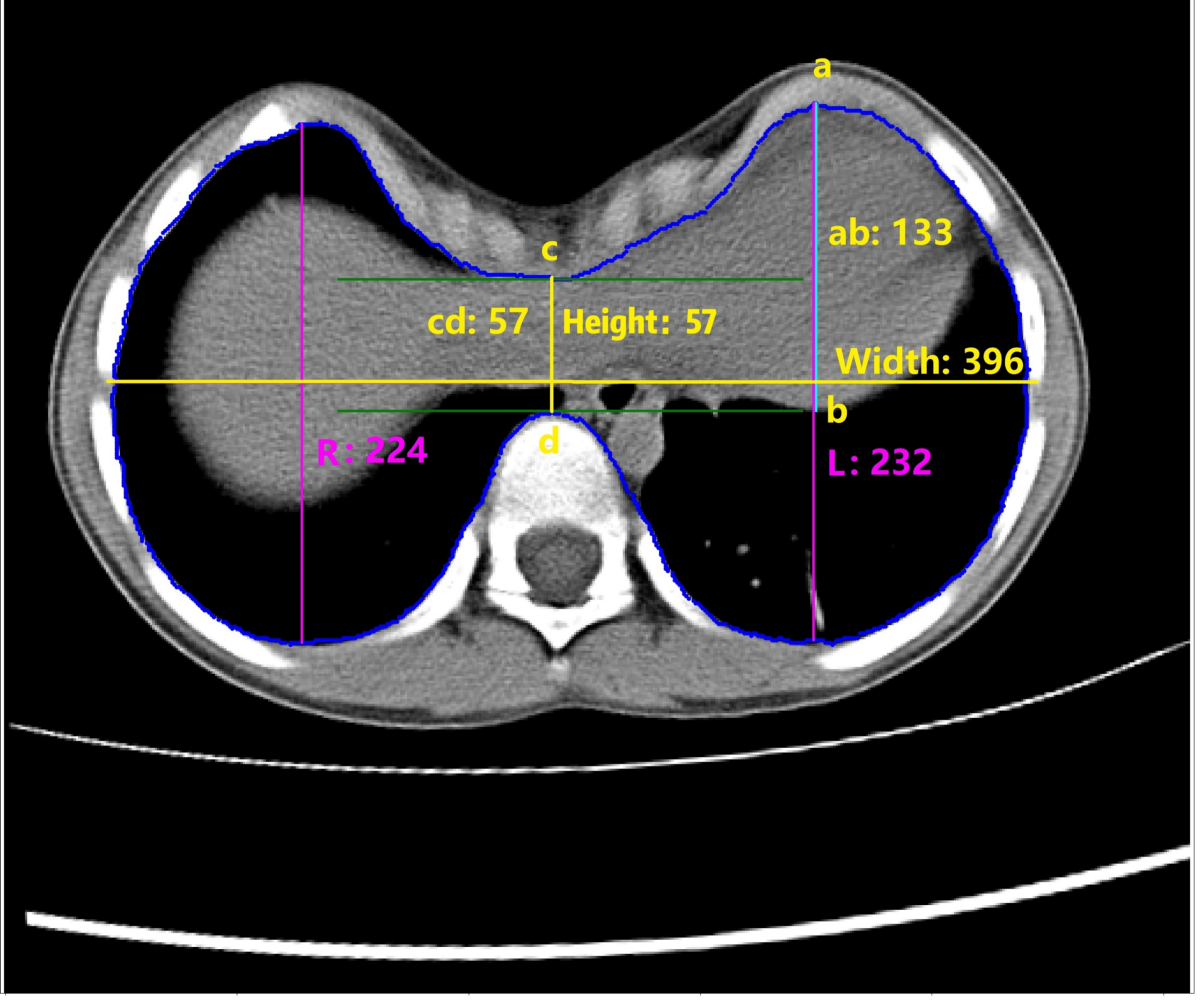
Automated pectus excavatum index calculation via U-Net segmentation. Height: the transverse diameter of the internal contour of the chest. Width: the anteroposterior distance of the internal contour. Haller index=width/height. "b" is the intersection extended from the tangent at the most anterior point of the spine on the internal contour and the vertical line through point "a." "c" is the lowest point on the anterior internal contour in pectus excavatum. "d" is the most anterior point of the spine on the internal contour. Correction index=((ab-cd)/ab)*100%. "L" and "R" represent the maximum anterior-posterior distances on the left and right sides of the internal contour, respectively. Asymmetry index=R/L. All distances are in centimeters.

With the U-Net segment model trained, the inner contour of each CT image could be obtained from the predicted results of the model. Therefore, the distances to calculate PE indices can be easily taken on the inner contours, and the respondent indices, including Haller index [11], correction index [3], and asymmetry index [12], can be calculated automatically on axial images with the deepest point of the sternum for each patient (Figure 2).

**Figure 3:**
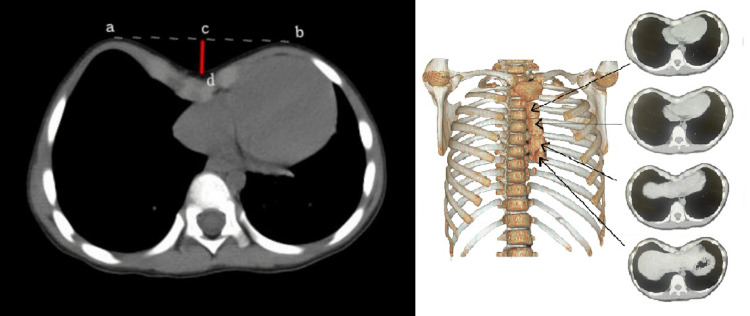
Selection of critical CT slices for severe pectus excavatum assessment. Left panel: How was the image of interest in a set of cross-sectional series selected. ab: a tangent line connecting the highest point of the front chest on both sides. cd: a perpendicular segment from the deepest point of the concave to line ab. Right panel: different levels of axial images were chosen for model building. Note only four levels of images are shown.

In fact, there are dozens of CT images for the set of axial view CT images of a specific patient. To efficiently segment the CT images by focusing on the examination of the most severe images, we can identify the image around the deepest depression. For each of the images of a single patient, the following selection steps were taken: on the outer contour, an assistant tangent line across the two highest points of the front chest on both sides is drawn; the distance from the deepest point of the sternum to the auxiliary line was calculated. The target image with the longest distance was selected; in the sorted images, we selected six images for internal contour delineation, including three images above the target and three images below it. In Figure 3, note that only four images are shown.

**Figure 4:**
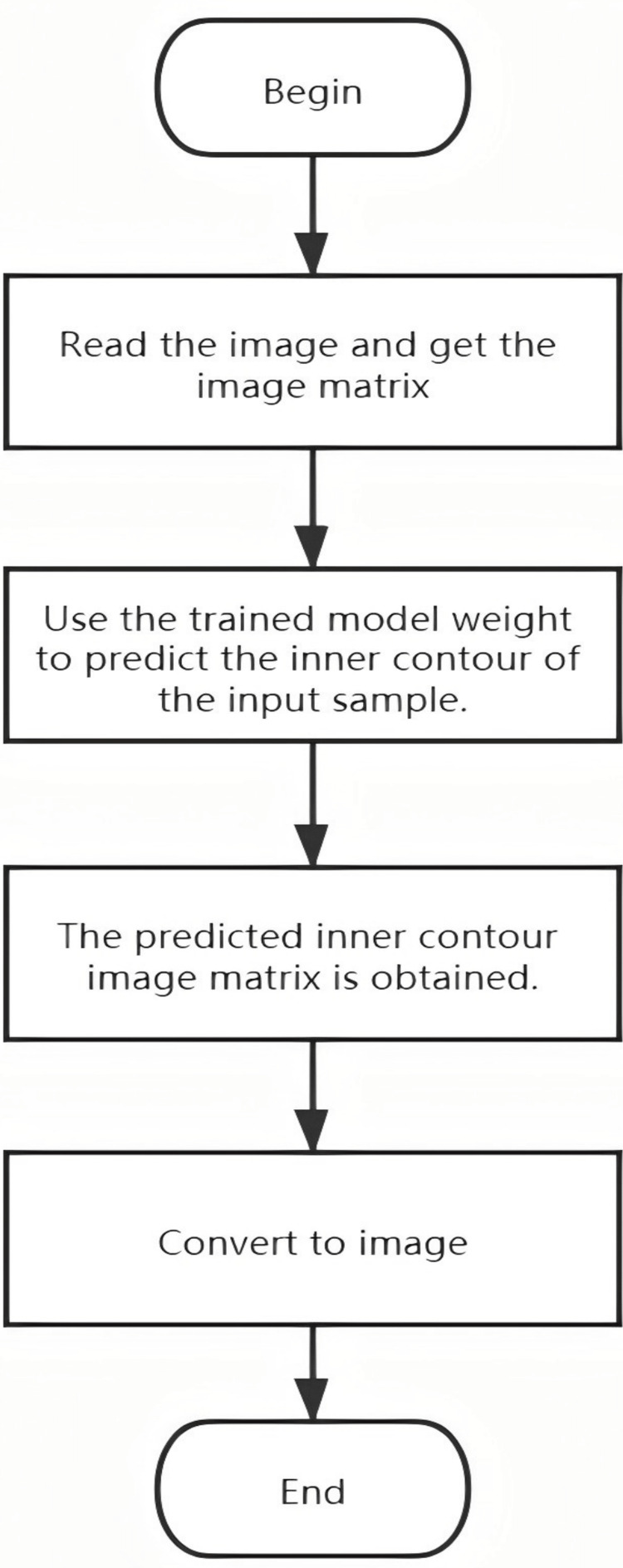
Automated multi-index measurement for pectus excavatum evaluation. Flowchart showing the segmentation process of internal chest contour from an axial image of chest CT.

Once the images around the deepest depression were selected and the internal contour was delineated, the points should be located easily for different distance measurements according to the definition of different indices. The following three indices were measured automatically: Haller Index, correction index, and asymmetry index. Figure 4 shows the automatic process of multi-index measurement for pectus excavatum.

To test the performance of our method, the results were compared with the experts' ground truth. Three radiologists measured each image to decrease interobserver variation and errors. For every person, each measurement was repeated, and an average of the two measurements was recorded as the final figure. If the difference between the index and mean of the index measured by the three radiologists was larger than 10% of the mean (defined as an error), then the result among the three with the largest difference was to be replaced by measurements of the fourth person, which was called “correction by the fourth person.” Therefore, the automatic measurement results were compared twice, with manual results before and after correction by the fourth person, respectively.

Statistical analysis was performed using Stata 16.0 (STATA Corp, College Station, TX) and Medcalc 18.2.1 (Medcalc Software bvba, Ostend, Flanders, Belgium). The data obtained were examined for normal distribution. Categorical data were present as frequencies, while continuous data that did not follow normal distribution were presented as median and interquartile range. The Pearson chi-square test was used to compare categorical data. The intra-class correlation coefficient was used to determine the consistency between manual and artificial intelligence (AI) measurements. Furthermore, the Bland-Altman plot was used to visualize their correlation. A p<0.05 was deemed statistically significant.

## Results

To evaluate the performance of the automatic segmentation, we compared its accuracy with the ground truth (manual segmentation). Specifically, we used the Dice similarity coefficient (DSC) and the intersection over union (IoU). The DSC measures the similarity between the automatic and manual segmentations. The IoU is used to measure the similarity between two sets. A total of 550 images from 94 patients were used for model training. For our model, the average DSC value was 0.905, and the average IoU value was 0.826.

**Figure 5:**
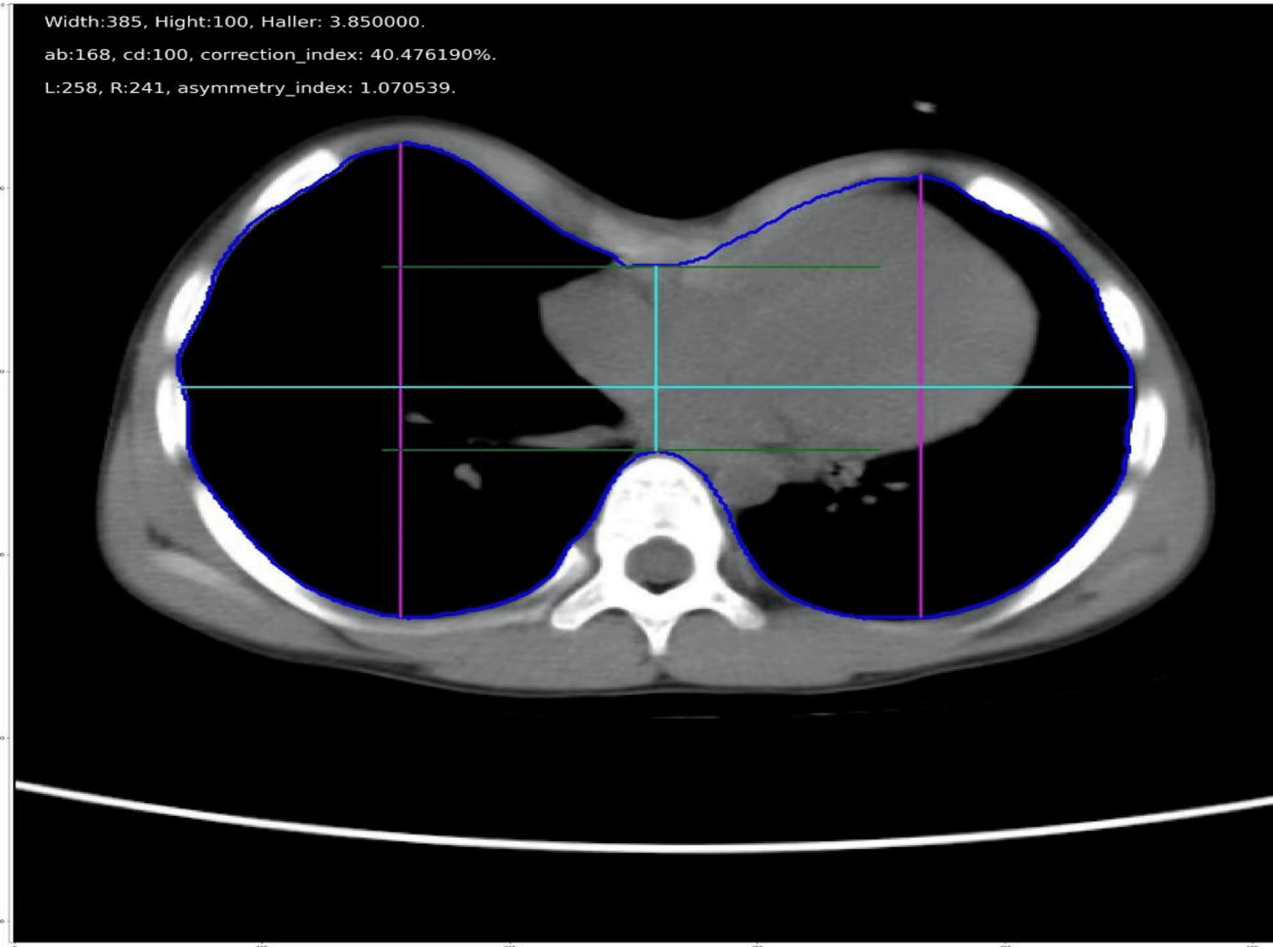
Accuracy and efficiency validation of automated pectus excavatum index measurement. An example of data and image output. Indices are shown in the upper-left corner of the image. All distances are in centimeters.

For the improvement of efficiency, the time used for the three indices was measured by experts, and our method was calculated for comparison. Specifically, human experts measured 20 patients with PACS CT software by clicking the mouse, and the average time of about five minutes for one patient was taken as the human efficient performance. As a result, our automatic system was able to select the image of interest with internal contour delineated and three indices measured in less than five seconds for one patient. Figure 5 shows an example of automatic output.

A total of 164 sets (one set for each patient) of images were used for validation. For each set, three indices, including Haller, correction, and asymmetry indices, were measured, including a total of 492 measurements for each radiologist or AI. An error rate counted as wrong measurements divided by all measurements (492) as high as 15.9% was seen for manual measurement before fourth-person correction, and it dropped to 4.1% after correction (p=0.00) (Table 1). For AI error, there was no statistical difference before and after correction, being 8.7% versus 8.5% (p=0.91) (Table 2). When comparing total error rates between manual and AI methods, it was significantly higher (p=0.003) in the manual group, yet lower (p=0.007) after being corrected by the fourth person (Table 3).

**Table 1:** Manual measurement error rates before and after correction by the fourth person. ^a^Measurement error was defined as more than 10% deviation from mean and measurement error rates were presented as number of errors/number of measurements (percentage). *Statistical significance. Pearson chi-square test was used for statistical analysis.

	Measurement error rates^a^		Chi-square value	p-value
	Before the fourth person correction	After the fourth person correction		
Haller index	26/164	7/164	14.43	0.002*
Correction index	50/164	13/164	28.08​	0.000*
Asymmetry index	2/164	0/164	2.01	0.16
Total error	78/492 (15.90%)	20/492 (4.10%)	23.62​	0.000*

**Table 2:** AI measurement error rates before and after correction by the fourth person. ^a^Measurement error was defined as more than 10% deviation from mean and measurement error rates were presented as number of errors/number of measurements (percentage). Pearson chi-square test was used for statistical analysis.

	Measurement error rates^a^		p-value
	Before correction	After correction	
Haller index	6/164	6/164	1.00
>10% of mean	8/164	11/164	0.50
Combined	14/164	17/164	0.61
Correction index	17/164	14/164	0.61
>10% of mean	9/164	10/164	0.65
Combined	26/164	24/164	0.82
Asymmetry index	2/164	1/164	0.57
>10% of mean	1/164	0/164	0.32
Combined	3/164	1/164	0.32
Total	25/492	21/492	0.58
>10% of mean	18/492	21/492	0.65
Combined	43/492 (8.7%)	42/492 (8.5%)	0.91

**Table 3:** Comparison of total error rates between manual and AI methods. ^a^Measurement error was defined as more than 10% deviation from mean and measurement error rates were presented as number of errors/number of measurements(percentage). *Statistical significance. Pearson chi-square test was used for statistical analysis.

	Total measurement error rates^a^		Chi-square value	p-value
	Manual	AI		
Before correction	78/492	43/492	9.67	0.003*
After correction	20/492	42/492	7.29	0.007*

**Figure 6:**
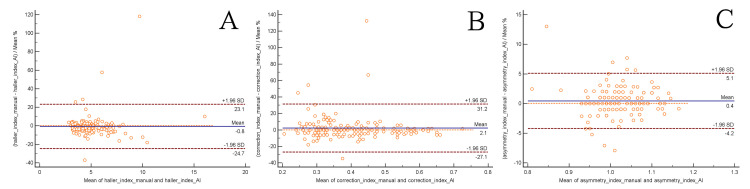
Inter-method agreement analysis of pectus excavatum indices using ICC and Bland-Altman. The Bland-Altman plots show good consistency between panel (A): manual and AI, the Haller Index; panel (B): manual and AI, the correction index; panel (C): manual and AI, the asymmetry index. ICC: intra-class correlation.

The statistical results by intra-class correlation coefficient are shown in Table 4. The correlation coefficient was 0.83 for the Haller index, 0.86 for the correction index, and 0.92 for the asymmetry index (all p values <0.01). The Bland-Altman plots show good consistency between manual and AI measurements, with average differences between the two methods being -0.8%, 2.1%, and 0.4%, for the Haller index, the correction index, and the asymmetry index, respectively (Figure 6).

**Table 4:** Intra-class correlation coefficient for each index between AI and manual methods. ICC: intra-class correlation.

	ICC (individual)	p-value
Haller index	0.83	<0.01
Correction index	0.86	<0.01
Asymmetry index	0.92	<0.01

## Discussion

The quantification of pectus excavatum severity is routinely assessed using CT and/or MRI scans. Evaluation of the Haller index in the PACS system is usually performed manually [11]. Over the past two decades, additional indices, such as the correction index and the asymmetry index, have been proposed for more detailed quantification. While the Haller index is worldwide used and accepted as the gold standard, its confidence to assess the severity of the deformity will be discussed more controversially, and alternative indices have been used more with an increasing frequency [13]. Using different indices may help to evaluate the severity and complexity of patients’ chest wall deformity more objectively and accurately, which is important for counseling as well as an effective treatment. For example, the correction index has better discriminable power than the Haller index [3,13], and the asymmetry index can quantify the asymmetry of the deformity [14], which cannot be assessed by the Haller index.

Although alternative methods have been introduced, including physical caliper [15,16], optical scanning [17-19], or two combined [20] instead of direct measurement on medical images, all were performed by hand. Measurement of multiple indices by hand is time-consuming, and the accuracy is observer-dependent. The most commonly used is the measurement of the Haller index at the level with the deepest concavity, which dictates a review of all cross-sectional images of the whole chest to decide the most depressed level. Sesia et al. suggested to use different standardized levels for measurement, which are located at the caudal end of corpus sterni to assess the Haller index, and at the sternomanubrial junction for asymmetry [14]. Since each patient's concave in the ventral chest wall is different in contour and level, and the chest width-to-depth ratio varies with age and thoracic levels [21], we consider an individualized level for measurement as more appropriate. This method necessitates many more measurements and is time-consuming, not to mention that even by an experienced technician or radiologist, low reliability and reproducibility is inevitable. This is the reason why we explored using a deep learning method to improve this process.

For automatic process imaging, segmentation is a fundamental and challenging problem for computer vision, aiming to partition an image in a meaningful way so that different objects can be localized, distinguished, and measured. In medical image processing, this is vital for further clinical analysis, diagnostics, treatment planning, and measuring. High precision and efficiency are typically required in medical image segmentation. Recently, segmentation methods based on deep convolutional neural networks have been developed for various medical imaging modalities, including MRI, CT, and X-ray, showing promising results and availability for clinical application [22]. U-Net network is an image segmentation network based on a convolutional neural network, which is mainly used for medical image segmentation. Its features can save manual annotation effort in a wide variety of image-related processes, making it very suitable for automated large-scale visual tasks. The network was originally proposed for cell wall segmentation [23] and has shown results comparable in quality to manual annotation. Furthermore, it has excellent performance in lung nodule detection, blood vessel and cartilage, and meniscus extraction [24-26].

In this study, we used an AI-based method to automatically take measurements one by one or in batches, making it more efficient and less observer-dependent. There have been only a few studies in terms of automatic measurement of pectus excavatum indices. Kim et al. [27] have reported a method based on the modified gradient vector flow snake method, which is not completely automatic. The algorithm used in their study requires setting the initial points of boundary extraction manually to initiate segmentation. Also, they have not mentioned how to automatically select the image of interest from a set of axial images. Another research group reported the qualification of PE based on the VGG16 deep learning model, which could not provide the calculation of the indices [28]. Silva et al. [29] report a similar method to ours for quantification analysis of PE. They, using the model, identified eight key points for further calculation. In comparison, our method tried to segment first the inner contour of the axial chest CT image and then calculate the indices, which we think is more accurate and conforming to the clinical workflow of manual measurement.

Our results have shown significant interobserver variation when measuring by hand. Before bringing in the fourth person's correction, the error rates, defined by more than 10% deviation from the mean, were as high as 15.9%. The selection of the image of interest and the measurement of a distance from point to point, among others, all have contributed to manual variations or errors. By contrast, it was robust to use AI-based automatic measurement. The identical image set input would output the same results regardless times of measurement, avoiding interobserver variation and human error. Moreover, it is much faster and more efficient than manual measurement. In terms of consistency between the two methods, the U-Net model has been shown to produce results with good accuracy, evidenced by high correlation coefficients and the Bland-Altman plots. This warrants its value in clinical application, though there were still some outliers, as shown in Bland-Altman plots. This automatic method may play an assistant role in conjunction with manual measurement to enhance efficiency and accuracy in a clinical setting or for research purposes.

Limitations

The present study has several limitations. First, the relatively small and homogeneous pediatric cohort (predominantly severe PE cases) may limit generalizability to mild-to-moderate PE populations. Second, our training and validation data were derived from a single institution using CT scans from a specific scanner (Philips), which restricts applicability to images acquired with other CT manufacturers or protocols. Third, the oldest patients in our cohort were adolescents (≤17.9 years), and the model's performance in adults, particularly females with developed breast tissue, remains unverified.

## Conclusions

The automatic method using U-Net model segmentation is a reliable approach to the automatic measurement of PE severity and could be considered for routine use in the clinical setting for a more reliable assessment of severity and degree of PE. More validation is needed to generalize its use to a wider population and other imaging work-up environments.
